# CRISPR-mediated accelerated domestication of African rice landraces

**DOI:** 10.1371/journal.pone.0229782

**Published:** 2020-03-03

**Authors:** Elia Lacchini, Edward Kiegle, Marco Castellani, Hélène Adam, Stefan Jouannic, Veronica Gregis, Martin M. Kater

**Affiliations:** 1 Department of Biosciences, University of Milan, Milan, Italy; 2 University of Montpellier, DIADE, IRD, Montpellier, France; Ecole Normale Superieure, FRANCE

## Abstract

African *Oryza glaberrima* and *Oryza sativa* landraces are considered valuable resources for breeding traits due to their adaptation to local environmental and soil conditions. They often possess superior resistance to endemic pests and tolerance to drought and nutrient deficiencies when compared to the “imported” high production Asian rice varieties. In contrast, “domestication traits” such as seed shattering, lodging, and seed yield are not well established in these African landraces. Therefore, the use of these African varieties for high production agriculture is limited by unpredictable yield and grain quality. We are addressing this shortcoming by developing protocols for genetically transforming African landraces to allow the use of CRISPR-Cas mediated breeding approaches. Here we use as proof of concept the cultivated African landrace Kabre to target selected known “domestication loci” and improve the agronomic potential of Kabre rice. Stable genetic transformation with CRISPR-Cas9-based vectors generated single and simultaneous multiple gene knockouts. Plants with reduced stature to diminish lodging were generated by disrupting the *HTD1* gene. Furthermore, three loci shown to control seed size and/or yield (*GS3*, *GW2* and *GN1A*) were targeted using a multiplex CRISPR-Cas9 construct. This resulted in mutants with significantly improved seed yield. Our study provides an example of how new breeding technologies can accelerate the development of highly productive African landrace rice varieties, an important advancement considering that Africa is a hotspot for worldwide population growth and therefore prone to food shortage.

## Introduction

Rice is the primary food source for half of the world’s population [1]. The genus *Oryza* is comprised of 23 species, two of which, *Oryza sativa* and *Oryza glaberrima*, have been systematically cultivated as a food crop [2]. *O*. *sativa* and its main two subspecies *indica* and *japonica*, were independently domesticated in the northeastern part of India and in South East China, respectively, roughly 10,000 years ago from wild *O*. *rufipogon* populations [3]. Cultivation of O. *glaberrima* began only 3,000 years ago around the upper Niger River Delta in West Africa, following differentiation from its wild ancestor *O*. *barthii* [4].

*O*. *sativa* varieties were introduced into West Africa by European colonizers around the 15^th^ and 16^th^ centuries, when trade between Africa and India was flourishing [5], and over time supplanted the cultivation of *O*. *glaberrima*, the native domesticated rice species. Many of these *O*. *sativa* varieties were subjected to human selection and evolved into landraces with adaptations beneficial under local conditions, but were never subjected to intensive and systematic breeding [6]. In Africa, both *O*. *sativa* and *O*. *glaberrima* accessions are grown using low-input, minimally mechanized agriculture, intended for local consumption [5] [7] [8]. The African landraces have been shown to carry several interesting traits that could empower sustainable and less demanding agricultural production. Valuable features present in African landrace germplasm were neglected for many years, as breeding programs focused on increasingly productive Asian rice varieties. Among these untapped traits are weed competitiveness, photoperiod insensitivity, and resistance to biotic and abiotic stresses [6] [9]. On the contrary, low yield, shattering and lodging are negative traits often present in African landraces that are lost in modern Asian rice varieties [10].

Since the *O*. *sativa* genome was published in 2002 [11] many yield-related loci repeatedly selected during domestication have been identified, mapped and functionally characterized [12]. Together with recent advances in CRISPR-Cas9 genome editing technology [13] [14], this knowledge creates unprecedented opportunities for crop improvement. A good example is the work done by Zhou and colleagues [15] who obtained a significant yield increase in *O*. *sativa* by simultaneous introduction of three trait related QTLs. To demonstrate that CRISPR technology has the potential to accelerate improvement of African landraces, we are developing transformation protocols for both African *O*. *glaberrima* and *O*. *sativa* landraces. Here we used as a proof of concept the African *O*. *sativa* landrace Kabre and targeted well-known domestication loci that are known to control plant height and seed yield.

## Materials and methods

### Agrobacterium mediated transformation of African landraces

The callus transformation protocol was adapted from Toki [16] and fully described in Supplemental File 1.

### Genome editing and constructs

Single knockouts were obtained using plasmids kindly provided by Miao et al. [17], following the guidelines reported by the authors [17], pOs-sgRNA was used to clone a single gRNA targeting *HTD1*. Subsequently, using the Gateway cloning system the cassette was moved to the binary vector pH-Ubi-cas9-7 encoding for the Cas9 endonuclease and hygromycin resistance as plant selection marker.

Multiple knockouts were made using pRGEB32, provided by Yinong Yang (Addgene plasmid # 63142) [18].

Four gRNAs of 20 nt, one for each gene to be disrupted *HTD1*, *GN1A*, *GS3* and *GW2*, were designed using the online tool CRISPR-P v2.0 (http://crispr.hzau.edu.cn/CRISPR2/). gRNAs proposed by the software were manually screened and selected based on their proximity to the start codon and avoiding potential off-targets.

### Targeted loci

Since Kabre was indicated to be an *O*. *glaberrima* accession both by the National Small Grain Collection (USDA) repository and in a previous publication [19], we initially designed the gRNA matching *HTD1* using the *O*. *glaberrima* orthologous gene sequence (ORGLA04G0179300) identified by performing a blast-search with the *O*. *sativa* locus (OS04G0550600). Prior to proceed with plant transformation in order to ensure a perfect sequence match between the *O*. *glaberrima* annotated gene and Kabre, the gRNA targeting location and neighboring region were cloned and sequenced using primers Osp_1332 and Osp_1333 (S3 Table). Primers Osp1209 and Osp1210 were used to create the gRNA for targeting *HTD1* (S3 Table).

As described in the results section, analysis of morphological traits and genotyping revealed that Kabre is most likely an *O*. *sativa* accession. For this reason, gRNAs used to target each of the three seed yield regulators *GS3*, *GN1A* and *GW2* were designed on the *O*. *sativa* loci Os03g0407400, Os01g0197700 and Os02g0244100, respectively. PCR amplification and sequencing of the Kabre gRNA-targeting regions was conducted using specific primers (S3 Table) to confirm sequence identity and the absence of mismatches.

Primers Osp1367 to Osp1372 (S3 Table) are the gRNAs for each of the three genes with adapters linked in order to assembly the multiplex CRISPR construct following detailed instructions published by Xie et al. [24] (pRGEB32 vector). Osp1584 and Osp1585, matching Cas9 of pRGEB32, were used to assess the presence of the transgene in plants transformed with the multiplex construct. Atp5706 and Atp5718 instead were adopted to screen for the presence of the transgene in plants transformed with the construct for a single gene knock out (i.e. Kabre *htd1*) as published by Miao et al [17].

### Genomic DNA extraction and PCR analysis

Genomic DNA (gDNA) was extracted following the protocol described in Supplemental File 1. All PCR reactions using gDNA were performed with GoTaq® (Promega) polymerase according to the company instructions.

### Genotyping

Genotyping of Kabre was conducted in order to assess whether it has an *O*. *sativa* or *O*. *glaberrima* genetic background. We amplified by PCR three different species-specific genetic markers *RM197*, *PROG1* and *S1* as described in the results section. PCR was performed using Terra^™^ PCR Direct Polymerase Mix (Takara) using a small piece of rice leaf directly in the reaction tube according to the Takara instruction manual. The following PCR condition were used: 98°C 2min, 35 cycles (98°C 10 sec, 60°C 15 sec, 68°C 30 sec), 68°C 10 min. Samples from *O*. *sativa* (IR64 and Gigante accessions) and *O*. *glaberrima* (CG14 and TOG5681 accessions) were used as control. All the primers used for this study are listed in S3 Table.

### Growth conditions

All the stages of tissue culture, except co-cultivation, were conducted under continuous cold fluorescent light at a temperature of 28° C. Once regenerants were ready for transplanting they were moved to soil and kept at 27°C in long day for about two months (18 hrs light). Later, plants were moved to short day conditions (14 hrs light) to induce flowering, after about two months rice seeds were harvested.

### Trait measurements

**Fig 1:**
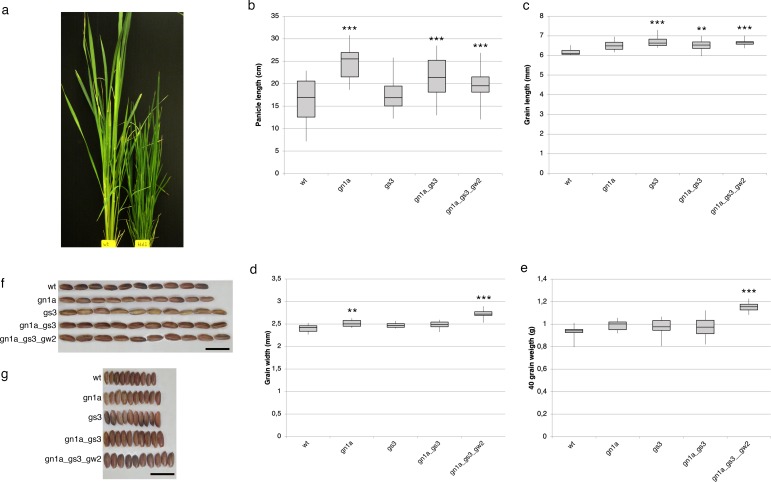
Phenotypical characterization of Kabre rice CRISPR-Cas mutants. (a) Kabre *htd1-1* dwarf mutant, on the right, showing excessive tiller production compared to the wild type, on the left. (b-g) Analyses of phenotypic variation of four traits between wild type (wt) and different mutant combinations. Box-and-whisker plots show the median of (b) panicle length, (c) grain length, (d) grain width, (e) 40-grain weight per genotype, upper and lower quartiles (box), and minima and maxima (whiskers). ANOVA and post-hoc Tukey HSD test were used, ***P < 0.01, **P < 0.05 for wt vs other genotypes comparison. The length (f) and width (g) of T_2_ seeds of wt, *gn1a*, *gs3*, *gn1a gs3* and *gn1a gs3 gw2* are shown. Scale bars = 1 cm.

Five different Cas9 T1 negative plants derived from *htd1-1* were selected and analyzed for the dwarf phenotype, composed by 3 and 2 plants having -7 and -17 bps homozygous deletion, respectively. Kabre mutants for seed traits were selected among the T2 generation. Seeds were measured for length and width using “SMARTgrain” a free program available online [20]. Six wild type, 10 *gs3* and 10 *gn1a* single mutants, 18 *gs3 gn1a* double mutants and 11 *gs3 gn1a gw2* triple mutant plants were analyzed taking 40 seeds from each plant; results are presented as average values (Fig 1). All selected mutant genotypes (e.g. single and double mutants) were equally composed in numbers by two different mutant alleles as described in the results section (S2 Fig). In contrast, T2 triple mutants were derived from a single T1 line and thus after segregation were comprised of 5 plants having either +1 insertion on *gn1a* and 6 plants carrying a -1 deletion on the same gene, while all the plants carried the same mutation on the two others loci *gs3* and *gw2* as reported in the Results section (S2 Fig). The statistical significance of differences was analyzed by variance (ANOVA) and post-hoc Tukey HSD.

## Results

### Transformation of the African landrace “Kabre”

CRISPR-Cas9 genome editing technology has great potential as a tool for plant improvement. However, one requirement for the use of this system is the ability to express the CRISPR guide RNA (gRNA) and Cas9 protein in plant cells. To investigate which African landraces can be used for *Agrobacterium*-mediated transformation, the regeneration capacity of embryogenic calli from different accessions was tested using a modified protocol published previously by Toki [16] (S1 File). All the African rice varieties that we tested were reported to be *Oryza glaberrima* rice species (Table 1).

**Table 1:** Oryza accessions selected for this study.

Accession ID	Origin	Institution	Regeneration
TOG 7196	Cote D'Ivoire	NSGC—USDA	-
TOG 7132	Senegal	NSGC—USDA	-
TOG 7125	Nigeria	NSGC—USDA	+
TOG 6946	Sierra Leone	NSGC—USDA	-
TOG 5548	Nigeria	NSGC—USDA	+
TOG 7275	Cameroon	NSGC—USDA	+
TOG 7261	Chad	NSGC—USDA	+
TOG 6231	Mali	NSGC—USDA	+
TOG 5681	Nigeria	IRD	+
CG14	Senegal	IRD	+
Ekassa	Guinea	NSGC—USDA	-
Sanganyan	Sierra Leone	NSGC—USDA	-
Kabre	Ghana	NSGC—USDA	+

Eight of the thirteen accessions that were selected for this study showed the ability to regenerate plantlets from calli cultured on hormone gradient plates (see examples in S1 Fig). The Kabre landrace from Northern Ghana was one of the most vigorously regenerating varieties and was selected for *Agrobacterium*-mediated transformation with CRISPR-Cas9 constructs. Notably, when grown in the greenhouse, Kabre plants showed phenotypes typical of landrace rice accessions but not modern cultivars, like elevated plant height, small seeds and small panicles. Kabre was thus a perfect candidate for our pilot experiment to show that by targeting specific “domestication” loci, fast improvement of landraces is possible.

Table 1 accessions were chosen based on their different geographical distribution across West Africa where *O*. *glaberrima* was domesticated. Accession identifiers can be used to retrieve online general information and features through the General Resources Information Network (GRIN–Global https://training.ars-grin.gov). TOG5681 and CG14 accessions were obtained from IRD (Montpellier, France). All the remaining accessions were obtained from the U.S.D.A. National Small Grains Collection (NSGC, Stuttgart, Arkansas, USA). In the last column those accessions that showed regeneration of plantlets from calli are indicated with “+”. The hormonal concentrations for which we obtained an optimal regeneration are reported in S2 Table.

### Improving plant height and tillering

African landraces are often lodging-sensitive due to their excessive height. In Asian rice, loss of function *oshtd1* mutants were reported to exhibit dwarfism and over-proliferation of tillers [21], making plants less lodging sensitive and able to produce a higher number of panicles per plant. *OsHTD1* (OS04G0550600), encodes a carotenoid cleavage dioxygenase. We performed a Genebank search to identify the *O*. *glaberrima HTD1* (*OgHTD1*) ortholog. The *OgHTD1* gene was identified on chromosome 4 (ORGLA04G0179300) sharing over 99% sequence homology with *OsHTD1*. For CRISPR-mediated mutagenesis a single gRNA matching the second exon of the open reading frame (575 bp downstream of the start codon) of *OgHTD1* was designed (S2 Fig). Subsequently embryogenic Kabre calli were transformed with the pH-Ubi-cas9-7 editing construct [17] and the two *htd1* mutants that were obtained contained bi-allelic mutations. *Htd1-1* harbored deletions of 17 and 7 bases and *htd1-2* deletions of 3 and 2 base pairs (S2 Fig).

Five Cas9 negative plants from the T1 progeny of *htd1-1* were analyzed. These plants displayed excessive tiller production and dwarfism (Fig 1A) in agreement with what has been previously shown in *O*. *sativa Nipponbare* [21] thus indicating potential use of this mutation to reduce sensitivity to lodging in this African landrace. As compared to the wild type *HTD1* locus which encodes for 609 amino acids, deletions of 17 and 7 base pairs would result in truncated proteins of 195 and 243 amino acids, respectively (S2 Fig). The obtained phenotypes suggest that CRISPR-mediated approaches could lead to important traits improvement in African rice landraces.

### Kabre has characteristics typical for *O*. *sativa*

**Fig 2:**
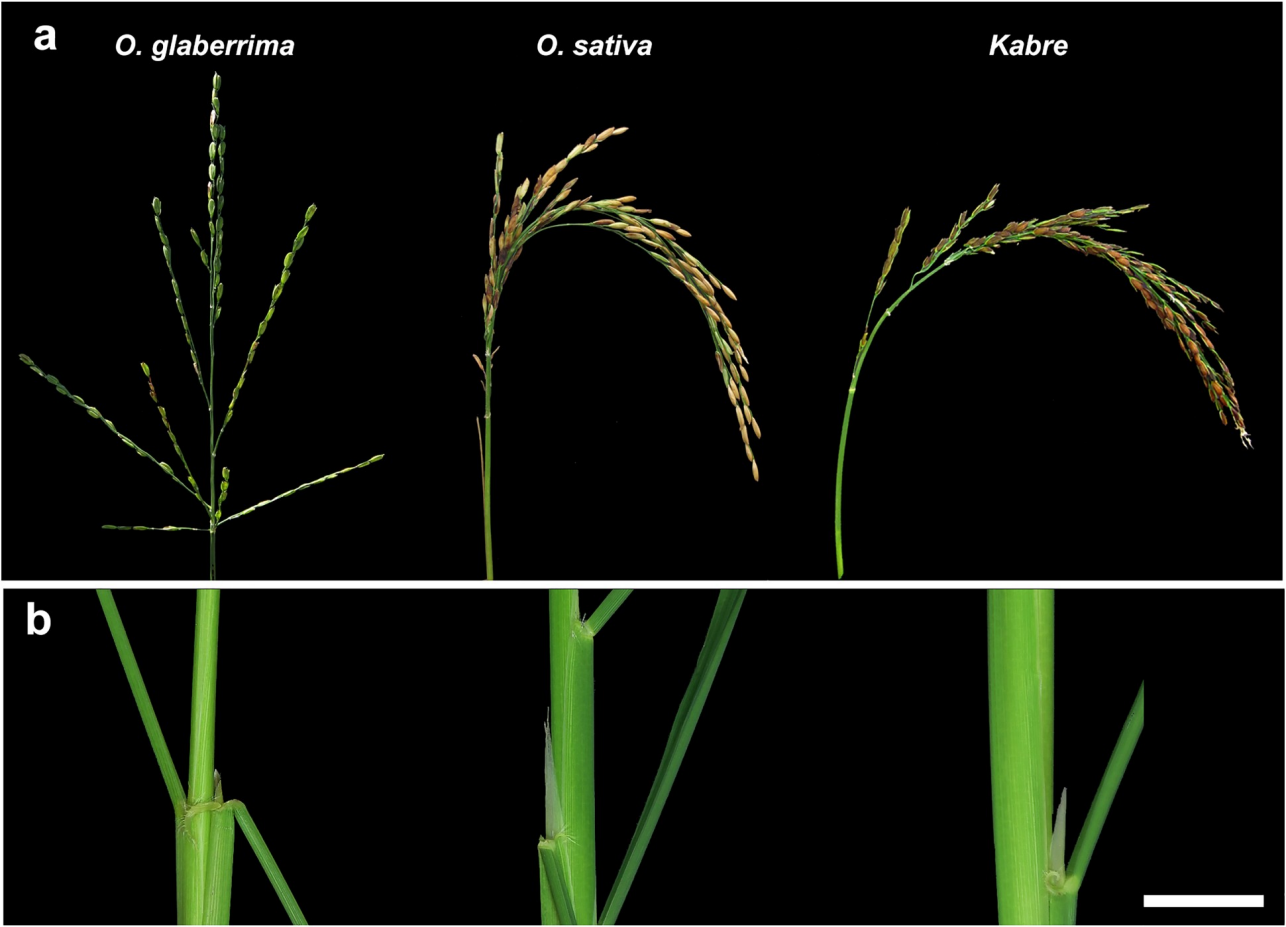
Comparative phenotypic features of Kabre accessions *with O*. *glaberrima* and *O*. *sativa*. (a) mature panicles, (b) close view of ligules. *O*. *glaberrim*a: TOG5681 accession; *O*. *sativa*: *indica* cv. IR64. Scale bar: 2 cm.

**Fig 3:**
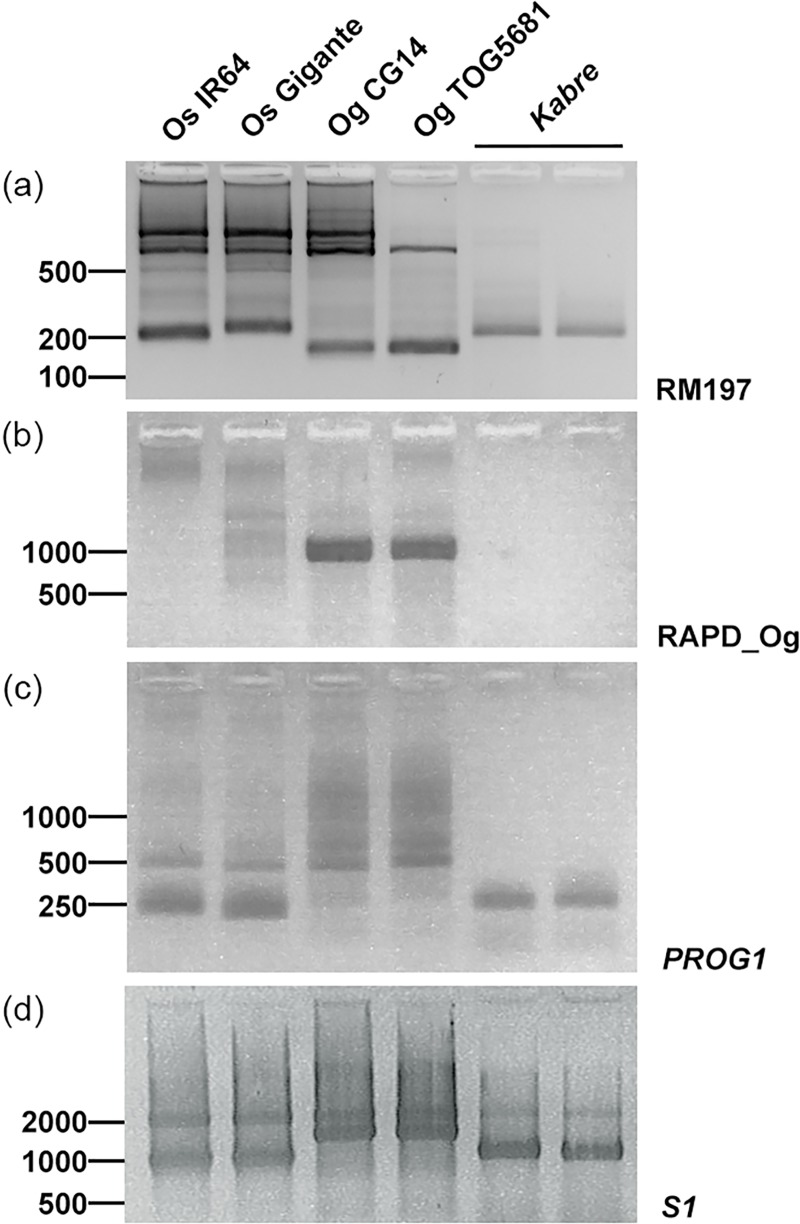
Genotyping of Kabre accession using markers discriminating *O*. *sativa* and *O*. *glaberrima* species. Genotyping was carried out using (a) the RM197 SSR marker (RM197), (b) a marker of the deletion around the *PROG1* gene in *O*. *glaberrima* (RAPD_Og), (c) *PROG1* gene specific primers, (d) *S*_*1*_ locus marker (*S1*). *O*. *sativa* cv. IR64, *O*. *sativa* cv. Gigante, *O*. *glaberrima* CG14, *O*. *glaberrima* TOG5681 were used as controls. The last two lanes represent two individuals from the Kabre accession. DNA marker sizes are indicated on the left (bp). The primers used for this study are listed in S3 Table.

When carefully analyzing the *htd1-1* and *htd1-2* mutant phenotypes we observed that the mutant and wild-type Kabre plants showed some typical *O*. *sativa* characteristics, despite being registered as an *O*. *glaberrima* species. The mature panicle was not erect and open as in *O*. *glaberrima* but more like in *O*. *sativa* (Fig 2A). Moreover, the ligule, the membranous appendage on the adaxial surface of a leaf, at the junction between the leaf sheath and the leaf blade was typical of *O*. *sativa* [22] (Fig 2B). Therefore, we decided to investigate the genetic background of Kabre in more detail and performed a genotyping experiment using different markers discriminating *O*. *sativa* and *O*. *glaberrima* species (Fig 3). Firstly, SSR marker analysis using the marker RM197 was performed according to Chen et al. [23]. Furthermore, the deletion of the *PROG1* gene and a deletion nearby this gene were investigated by PCR analysis since they are reported to be reliable species-specific *O*. *glaberrima* markers [8] [24]. Lastly, a marker specific to the *S*_*1*_ locus corresponding to a postzygotic reproductive barrier–associated locus between *O*. *sativa* and *O*. *glaberrima* was also used for the analysis [25]. As reference controls for the two different species genomic DNA extracted from *O*. *sativa* cv. IR64 and cv. Gigante and from *O*. *glaberrima* accessions CG14 and TOG5681 were used. All these analyses suggest that Kabre is an African *O*. *sativa* landrace.

### Multiple trait stacking for seed yield improvement in Kabre

To further investigate possible yield improvements in African rice landraces by simultaneously stacking multiple agronomic traits selected during domestication in Asian rice, three genes, *GRAIN NUMBER 1A* (*GN1A*), *GRAIN SIZE 3* (*GS3*) and *GRAIN WEIGHT 2* (*GW2*) [12] were selected for mutagenesis. These loci are known to be major QTLs for negatively regulating plant yield. *GN1A* (*OsCXK2*), artificially selected during domestication, encodes a cytokinin oxidase/dehydrogenase that negatively controls panicle length and consequently spikelet number [26]. *GS3* encodes a Gγ protein subunit containing a transmembrane domain spanning the plasma membrane and is implicated as a negative regulator of seed length and weight in several cultivars [27]. Lastly, *GW2* encodes a RING-type E3 ubiquitin ligase and plays a role as negative regulator of grain girth and weight [28].

One gRNA for each of the three target genes was designed based on the *O*. *sativa japonica* genome sequence [11]. gRNAs showing ideal features in terms of target specificity and GC content were designed to match the first, fifth and second exon of the *GN1A*, *GS3* and *GW2* locus, respectively (S2 Fig). Subsequently the target sequences were verified by sequencing the gRNA matching regions for the three selected loci in the Kabre background to verify the absence of any sequence mismatch (S3 Table). The gRNA sequences were then cloned into the CRISPR multiplex vector pRGEB32 [24]. Following transformation, 76 plants were identified as Cas9 positive by PCR screening. Disrupted loci were verified by sequencing. Four classes of mutants were obtained in T_0_: 3 *gn1a* and 19 *gs3* single mutants, 38 *gn1a gs3* double mutants, and 16 *gn1a gs3 gw2* triple mutants. Among the 16 triple mutants, only one plant carried the *gw2* mutation in homozygosity, all the others were heterozygous for *gw2*. All the other plants (single and double mutants) had the targeted loci mutated in homozygosity or carried bi-allelic mutations suggesting that the gRNA targeting *GW2* had the lowest efficiency. In the T_1_ generation, Cas9 negative plants were selected and grown for phenotypic analysis in the T_2_ generation. In total, six wild type (WT), 10 *gs3* and 10 *gn1a* single mutants, 18 *gs3 gn1a* double mutants and 11 *gs3 gn1a gw2* triple mutant plants were analyzed. Of these lines we selected two independent mutant lines for further phenotypical analysis. In T_2_ selected single mutant *gs3* lines carried either a +1 insertion or a -2 deletion, predicted to result in a protein matching the wild type only for the first 150 amino acids and lacking in-frame stop codon thus likely resulting in an aberrant transcript and leading to non-sense mediated decay [29]. Single *gn1a* mutants analyzed instead carried either a -2 bps deletion or a +1 bp insertion, in both cases the resulting protein were predicted to match wild type only for the first 86 amino acids and contained premature stop codons. *gn1a gs3* double mutant lines selected for phenotypic analysis of seed features either carried a -2 or -4 bps deletion in the *gs3* locus, the latter possibly translating in an aberrant protein that matched the wild type for the first 150 amino acids, while both genotypes had homozygous -2 bps deletion for *gn1a*. Finally, Cas9 negative plants were selected among the segregant T_2_ progeny obtained from a T_1_ triple mutant carrying a -2 bps deletion at the *gs3* locus, a biallelic +1/-1 bp mutation for *gn1a* and a -1 homozygous deletion on the *gw2* locus. The latter was predicted to translate to a non-functional truncated protein of 89 amino acids.

The *gn1a* single mutants showed an important increase in panicle length of approximately 49% relative to wild type. Interestingly, combining the *gn1a* mutant with *gs3* and *gw2* partially attenuated this phenotype, even though the overall panicle length increase was maintained (Fig 1B). Grain length and width were measured with SMARTGrain software [20] (Fig 1C and 1D). Analysis of seed length showed that *gs3*, *gs3 gn1a* and *gs3 gn1a gw2* mutants all produced longer seeds compared to wild type (see also Fig 1F). Among the four classes of mutants, seed width was slightly increased in *gn1a* and drastically increased in the triple mutant. Moreover, the *gn1a gs3 gw2* mutant produced seeds with a significantly enhanced weight of 24% compared to wild type (Fig 1E). This analysis confirmed that, among the three genes, mutating *GW2* in Kabre caused the most significant increase in seed width and weight (Fig 1D, 1E and 1G).

## Discussion

This study shows that CRISPR-Cas9 mediated mutagenesis promises to be an important tool for improvement of local African landraces. The development of transformation protocols or other technologies to introduce the CRISPR-Cas machinery into living plant cells is therefore of great importance. Our regeneration experiments on hormone gradient plates using 13 different African rice accessions revealed a high variation in regeneration capacity. Therefore, most likely the bottleneck to genome edit different rice accessions adapted to local conditions will at this moment likely not be the CRISPR-Cas technology but more the development of transformation protocols.

We selected Kabre for its high regeneration capacity but especially because it is a typical African landrace with low yield and not subjected to modern breeding. Although being considered an *O*. *glaberrima* landrace by the U.S. National Germoplasm System (https://npgsweb.ars-grin.gov) as well as in previous studies [19], analysis of specific loci in its genome suggests that it is an *O*. *sativa* accession rather than being an *O*. *glaberrima*; nevertheless it has characteristics found in both species. Despite all markers used showed the same results that we obtained for the *O*. *sativa* control plants we cannot completely exclude that Kabre is the result of an introgression between *O*. *sativa* and *O*. *glaberrima*. Complete genome sequencing of Kabre might reveal its origin.

Recently, Zhou et al. [15] CRISPR edited the same three QTLs in Chinese elite rice varieties. In contrast to our observations, the authors showed that the *gn1a gs3 gw2* triple mutants overall have longer and bigger panicles than any other class of mutants for the three genes. It is likely that the difference in panicle length caused by the *gn1a* mutation is due to the genetic background of the rice accessions used. While Zhou and colleagues [15] aimed at a further boost in grain yield from already productive, highly selected, and likely genetically more homogeneous *O*. *sativa* varieties, our work was conducted in a more heterogeneous African landrace with a genetic background that was not selected for high productivity.

Importantly, our data show that the accelerated domestication approach by simultaneously stacking multiple mutant combinations through CRISPR-Cas technology provides a means for rapid domestication of African landraces that have traits making them more suitable for sustainable agriculture. Interestingly, by applying the latest CRISPR mediated base-editing technologies [30] [31] it will not only be possible to obtain harvest increases by knocking out negative regulators of yield, as shown in this study, but it will also allow the introduction of any kind of favorable mutation. Altogether if these tools can be introduced in plant cells, they will empower fast adaptation of orphan and neglected crops to facilitate their use in modern high yielding agriculture practices. Furthermore, improvement of ‘native’ local crops could empower farmers in Africa and elsewhere to grow familiar crops, already adapted to a particular niche, with greater productivity and environmental sustainability. This might well be important to take in consideration since Africa is expected to be the hotspot of population growth (www.un.org).

## Conclusions

Climate change and world population growth, especially in Africa, demands for more short-term solutions to increase crop yield and sustainability of agriculture. CRISPR-Cas based gene editing technology promises to contribute to find solutions, also due to the increased knowledge about gene functions obtained over the last 30 years. Here we demonstrate that knowledge transfer from high productive Asian rice varieties to local African landraces may facilitate accelerated improvement of these crops without losing their specific benefits for the growth of these plants under local conditions. An important requisite to be able to tap into the CRISPR-Cas toolbox is the introduction of this system into plant cells. We foresee a revival of scientists studying tissue culture practices to introduce stably or transiently engineered Cas proteins and gRNA into target plant cells for highly precise gene engineering *in vivo*.

## Supporting information

S1 Raw images (PDF)

S1 File Agrobacterium mediated transformation of African landraces, detailed protocol and genomic DNA extraction procedure. (PDF)

S1 Fig Regeneration of African rice accessions on hormone gradient plates. (PDF)

S2 Fig Graphical schematization of genomic location of target genes and gRNA position and edited loci of selected mutant genotypes. (PDF)

S1 Table Kinetin (KIN) and α-NaphtAlenic Acid (NAA) concentrations used in gradient plates to test regeneration conditions. (PDF)

S2 Table Optimal concentrations of Kinetin (KIN) and α-NaphtAlenic Acid (NAA) to regenerate African rice accessions. (PDF)

S3 Table Primers: Target locus and their use are reported. (PDF)
