# Supplementary material for: CRISPR-mediated accelerated domestication of African rice landraces
Source: PLoS One. 2020 Mar 3;15(3):e0229782. doi: 10.1371/journal.pone.0229782 (PMC7053755; doi:10.1371/journal.pone.0229782)
Supplement: S1 Raw images — (PDF) [file pone.0229782.s001.pdf]

TERRA DNA pol
98°C 2min
35 cycles 98°C 10 sec
60°C 15 sec
68°C 30 sec
68°C 10 min

1. **Os** IR64
2. **Os** Gigante
3. **Og** CG14
4. **Og** Tog581
5. **Kabre** KT2#1
6. **Kabre** KT1#1

1 2 3 4 5 6

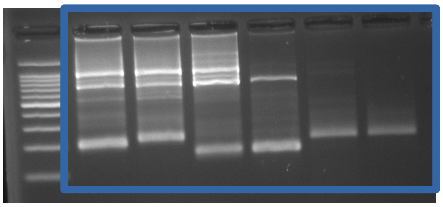

RM197

3% agarose gel

1 2 3 4 5 6

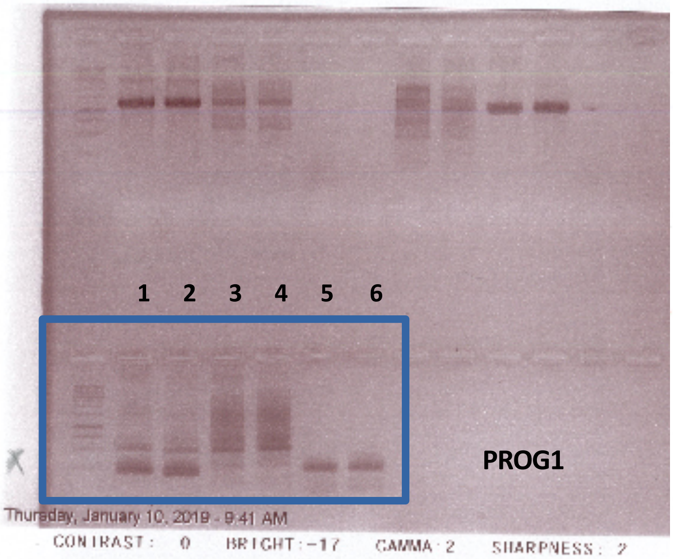

1 2 3 4 5 6

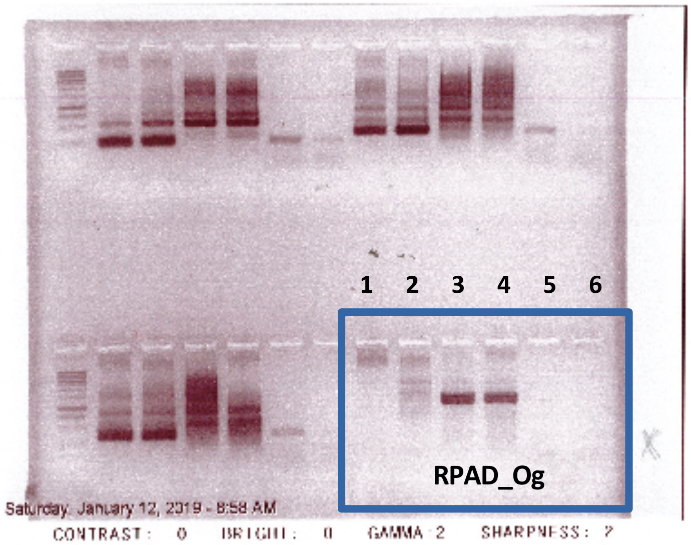

1,5% agarose gel

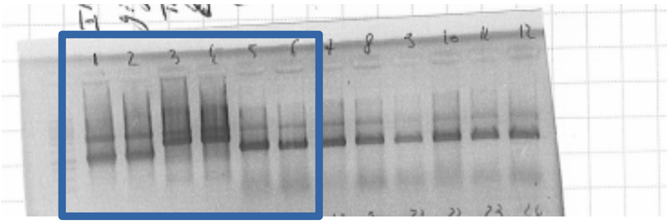

S1 locus

2% agarose gel

1. **1. *O sativa*** IR64
2. **2. *O sativa*** Gigante
3. **3. *O glaberrima*** CG14
4. **4. *O glaberrima*** Tog581
5. **5-12. *Kabre***

Part of the raw images used for the Figure 3
