# Supplementary material for: CRISPR-mediated accelerated domestication of African rice landraces
Source: PLoS One. 2020 Mar 3;15(3):e0229782. doi: 10.1371/journal.pone.0229782 (PMC7053755; doi:10.1371/journal.pone.0229782)
Supplement: S1 File — (PDF) [file pone.0229782.s002.pdf]

Supplemental File 1

Agrobacterium mediated transformation of African landraces

Callus trasformation protocol was adapted from Toki (1997).

Induction of embryogenic calli.

Surface sterilized seeds were sown, on Callus Induction Medium (CIM – N6D, Toki et al. 1997). As Toki et al., (1997) 2.8 g l-1 proline and 0.3g l-1 of casamino acids were found to positively affect callus production. Gelrite was used at 4g l-1. Medium was supplemented with 10% (v/v) filter-sterilized (0.2 µm mesh) Coconut Water (Foco®, Thailand) (Rangan 1974). The number of seeds per plate was critical for callus induction, optimally 5-6 seeds per plate. Petri dishes were sealed with ventilation tape (Micropore®, 3M). Calli were induced under continuous cold fluorescent light at 28°C. Depending on the cultivar and freshness of the seeds, calli derived from scutella are produced in about two weeks. To keep calli healthy and competent for transformation, calli were subcultured every 10 to 14 days.

Agrobacterium infection and co-cultivation

Agrobacterium tumefaciens (strain EHA105) cells transformed with binary vectors were grown for two days on YEP plates supplemented with Rifampicin (50 ug/ml) together with a second antibiotic according to the reistance carried on the binary vector. Two different vectors were used in plant transformation: pCas9 by Miao et al., (2013) for cloning of single gRNA while pRGEB32 as published by Xie et al., 2015 (Addgene plasmid # 63142), was used for gRNAs multiplexing. A single colony per each construct was picked and moved to 2 ml of liquid YEP with appropriate antibiotics and grown overnight. Agrobacterium (strain EHA105) cells were pelleted and resuspended in 100 ul AA-AS. Later resuspended Agrobacterium cells were diluted to reach a OD between 0.005 and 0.08. Typically, for O. glaberrima accessions we found OD 0.02 to be optimal.

Calli used in transformation were subcultured on fresh CIM for 4 days prior infection as in Hiei et al. (1994). Subcultured rice calli were then soaked in bacterial suspension, gently shaking, for 3 minutes (Toki 1997). Excess of bacteria was removed by pouring off the infection medium and blotting the calli on sterile Kimwipes or filter paper. Infected calli were placed onto N6-AS (Hiei et al., 1994) for co-cultivation. In order to avoid Agrobacterium overgrowth, sterile filter paper discs were placed between the calli and the surface of N6-AS medium. Calli and bacterial cells are co-cultivated at 22° C in darkness until bacterial masses can be seen with naked eyes and calli look translucent.

Selection of Transformed calli

Infected calli are moved to a sterile container and washed six or more times with sterile deionized water. For the final wash, carbenicellin or cefotaxime (500 mg l-1) was added to the washing solution. Calli were dried briefly on sterile Kimwipes or filter paper. Calli were moved to N6-P (N6 – S0) containing 500 mg l-1 carbenicellin or cefotaxime at 28°C under cold fluorescent light for about 10 days. Up to 30 calli were placed on each dish. Subsequently, calli were moved for two weeks to N6-P (N6 – S1) medium containing 25 mg l-1 hygromycin. Next, healthy, lightly colored resistant calli were transferred to a second round of selection with 50 mg l-1 Hygromycin (N6 – S2) for other two weeks.

Regeneration of Transgenic plants

Kinetin and NAA were tested at different concentrations in order to evaluate regeneration capacity of several Oryza accessions (Table 1). Data and hormone concentrations are reported in Table 3. Independent resistant calli were transferred to N6-R medium. Dishes contained 30 ml of N6-R and 6 calli per dish. Petri dishes were sealed with Micropore® tape to avoid condensation. Calli were kept at 28°C under continuous cold fluorescent light. Depending on the variety, green shoots should be seen within two weeks. Once shoots and roots are formed, plantlets were moved to Magenta© boxes containing MS-F medium. After 10 - 14 days plants were transplanted to soil.

Media

B5 micronutrients (1000x stock)

10g l-1 MnSO4 H2O, 0.75g l-1 KI, 3g l-1 H3BO3, 2g l-1 ZnSO4 7H20, 39mg l-1 CuSO4 5H20, 250 mg Na2MoO4 2H20, 50 mg l-1 CoCl2 6H20

Callus Induction and subculture (CIM - N6D)

N6D from Toki et al., (1997) added with 4 g l-1 Gelrite, B5 micronutrients, 10% filter sterilized Coconut Water, pH = 5.6

AA-AS liquid infection medium

4g l-1 N6 salts and vitamins, B5 micronutrients, 20g l-1 Sucrose, 10g l-1 Glucose, 1g l-1 Casamino acids, 100 μM Acetosyringone, pH = 5.6

N6-AS Co-cultivation

2N6-AS medium (Hiei et al., 1994) supplemented with 4g l-1 Gelrite, B5 micronutrients, 10% filter sterilized Coconut Water, pH = 5.6

N6-S0/1/2 Selection Medium

N6D from Toki et al. (1997) supplemented with 10% filter sterilized Coconut Water, 500 mg l-1Cefotaxime (S0), 25 mg l-1 Hygromycin (S1) and 50 mg l-1 Hygromycin (S2)

N6R Regeneration Medium

4g l-1 N6 salts and vitamins, B5 micronutrients, 1 g l-1 Casamino Acids, 25 g l-1 Sucrose, 25 g l-1 Sorbitol, 8 g l-1 Agarose Type I, Kinetin and NAA according to the accession (conventional values are Kinetin = 2 mg l-1, NAA = 0.05 mg l-1)

Gradient Plates

Regeneration of plantlets from calli is frequently considered the most critical step in tissue culture. In order to assess reliability and flexibility of our transformation protocol we used gradient plates to determine ideal concentration of hormones allowing proper development of plants roots and shoots.

Squared multi-well tissue culture plates were used for hormonal gradient; each well contained 5 ml of N6-R medium and a specific ratio of Kinetin and NAA.

Genomic DNA extraction

Rice leaf samples were ground in liquid nitrogen and resuspended in 400 uL of DNA extraction buffer (250 mM Tris HCl pH=7.5, 25 mM EDTA, 250 mM NaCl, 1% SDS). Samples were spin 13000 rpm on a bench top centrifuge at room temperature for 5 minutes to pellet cell debris. The supernatant was transfer to a new Eppendorf containing 0.8 volume of Isopropanol. Samples were spin at 13000 rpm for 15 minutes at room temperature to precipitate genomic DNA. Following removal of Isopropanol, the DNA pellet was washed by adding 250 uL of 70% Ethanol. Samples were spin at room temperature at 13000 rpm for 15 minutes. Ethanol was carefully removed avoid touching the DNA pellet and samples were let dry to ensure complete removal of Ethanol. Once dry genomic DNA was resuspended in 50 uL of sterile milliQ H2O containing 2 uL of RNAseA (10mg/ml). Samples were incubated at 37°C for 30 minutes to ensure removal of RNA contamination. Finally 1 uL was used for PCR-based applications.
