# Supplementary material for: CRISPR-mediated accelerated domestication of African rice landraces
Source: PLoS One. 2020 Mar 3;15(3):e0229782. doi: 10.1371/journal.pone.0229782 (PMC7053755; doi:10.1371/journal.pone.0229782)
Supplement: S1 Fig — (PDF) [file pone.0229782.s003.pdf]

Supplemental Figure S1

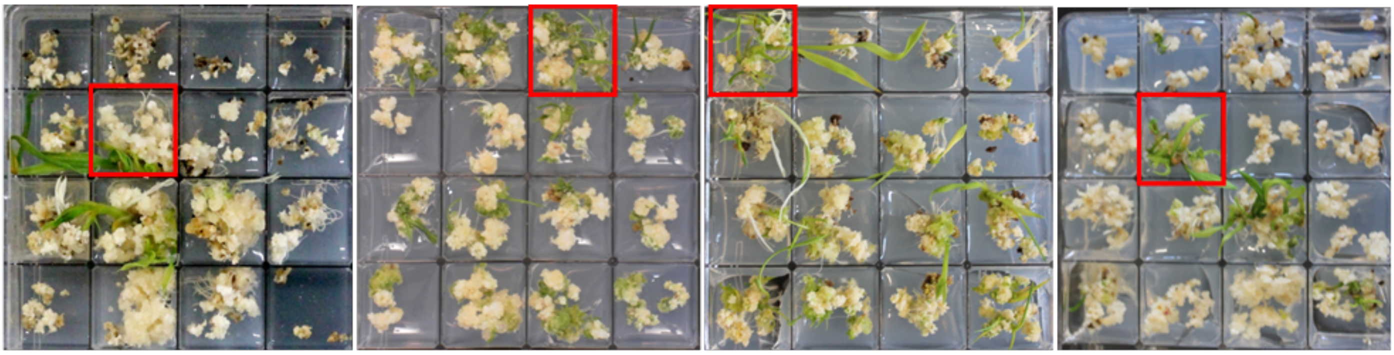

Examples of gradient plates for (left to right) TOG7275, TOG7261, TOG5548, CG14 optimal hormonal concentration ideal for root/shoot development are reported in the table below. Hormonal concentrations in each well are reported in Supplemental Table S1. Red Boxes highlight hormonal concentrations ideal for shoot/root regeneration of selected accession. Below are reported ideal hormonal concentration for plant regeneration of 8 selected accessions.
