# Supplementary material for: CRISPR-mediated accelerated domestication of African rice landraces
Source: PLoS One. 2020 Mar 3;15(3):e0229782. doi: 10.1371/journal.pone.0229782 (PMC7053755; doi:10.1371/journal.pone.0229782)

Supplemental Figure S2: A) exon – intron structure of target loci, gRNA sequences are reported for each locus, **B)** mutant genotypes considered in this study, gRNAs are highlighted in yellow, PAM sequence in bold

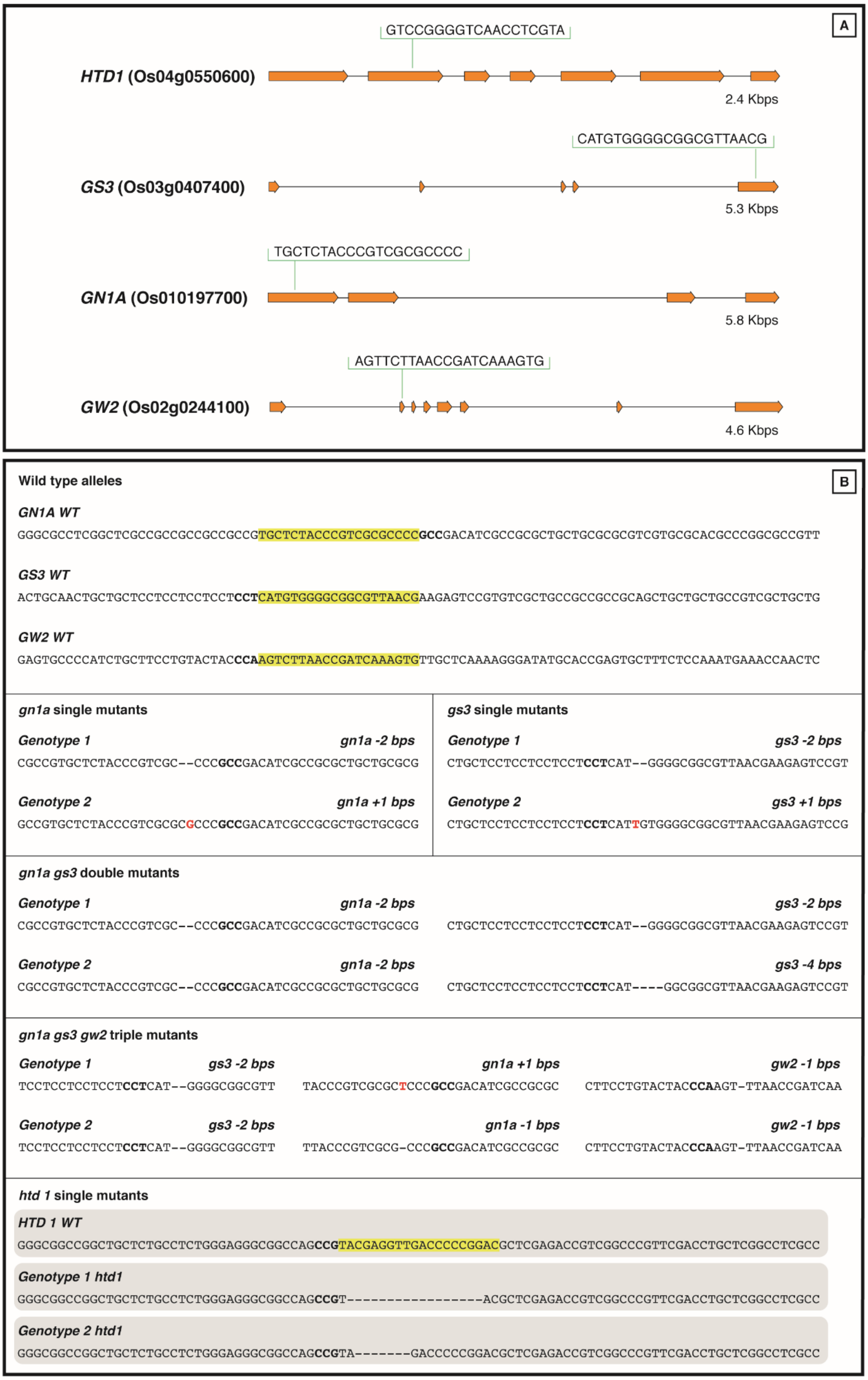

Supplement: S2 Fig — (PDF) [file pone.0229782.s004.pdf]
