# Supplementary material for: CRISPR-mediated accelerated domestication of African rice landraces
Source: PLoS One. 2020 Mar 3;15(3):e0229782. doi: 10.1371/journal.pone.0229782 (PMC7053755; doi:10.1371/journal.pone.0229782)
Supplement: S1 Table — (PDF) [file pone.0229782.s005.pdf]

Supplemental Table S1: Kinetin (KIN) and α-NaphtAlenic Acid (NAA) concentrations used in gradient plates to test regeneration conditions.

NAA (mg l^{-1})	Kinetin (mg l^{-1})			
	0.5	1	2	4
	0.01	0.01	0.01	0.01
	0.5	1	2	4
	0.05	0.05	0.05	0.05
	0.5	1	2	4
	0.5	0.5	0.5	0.5
	0.5	1	2	4
	1	1	1	1
