# Supplementary material for: CRISPR-mediated accelerated domestication of African rice landraces
Source: PLoS One. 2020 Mar 3;15(3):e0229782. doi: 10.1371/journal.pone.0229782 (PMC7053755; doi:10.1371/journal.pone.0229782)
Supplement: S2 Table — (PDF) [file pone.0229782.s006.pdf]

Supplemental Table S2: Optimal concentrations of Kinetin (KIN) and α-NaphtAlenic Acid (NAA) to regenerate African rice accessions

Accessions ID	NAA (mg l^{-1})	Kinetin (mg l^{-1})
Kabre	0.05	4
TOG6231	0.05	4
TOG7125	0.5	4
TOG7261	0.01	2
TOG7275	0.05	1
TOG5681	0.05	1
TOG5548	0.01	0.5
CG14	0.05	1
