# Supplementary material for: CRISPR-mediated accelerated domestication of African rice landraces
Source: PLoS One. 2020 Mar 3;15(3):e0229782. doi: 10.1371/journal.pone.0229782 (PMC7053755; doi:10.1371/journal.pone.0229782)
Supplement: S3 Table — (PDF) [file pone.0229782.s007.pdf]

Supplemental Table S3

Table of primers used in the study, target locus and use are reported

Primer ID	Sequence	Target gene	Use
OsP_1209	ggcaGTCCGGGGGTCAACCTCGTA	HTD1	gRNA + BsaI compatible ends (lowercase)
OsP_1210	aaacTACGAGGTTGACCCCCGGAC	HTD1	gRNA + BsaI compatible ends (lowercase)
OsP_1332	TCGATCTTGTAGTGCGCCAG	htd1	genotyping/editing check
OsP_1333	AAGGTGGGCAATGTGAAGGT	htd1	genotyping/editing check
OsP_1367	taggTCTCGACGCCGCCCCACATGgttttagagctagaa	Gs3_multiplex_frw	gRNA + adaptors Xie et al., (2015)
OsP_1368	cgGGTCTCAGCGTTAACGtgcaccagccggg	Gs3_multiplex_rev	gRNA + adaptors Xie et al., (2015)
OsP_1369	taGGTCTCGTGATCGGTTAAGACTgttttagagctagaa	Gw2_multiplex_frw	gRNA + adaptors Xie et al., (2015)
OsP_1370	cgGGTCTCAatcaaagtgtgcaccagccggg	Gw2_multiplex_rev	gRNA + adaptors Xie et al., (2015)
OsP_1371	taGGTCTCGGACGGGTAGAGCAgttttagagctagaa	Gn1a_multiplex_frw	gRNA + adaptors Xie et al., (2015)
OsP_1372	cgGGTCTCACGTCGCGCCCCtgcaccagccggg	Gn1a_multiplex_rev	gRNA + adaptors Xie et al., (2015)
OsP_1578	TCCGCCTCCTTCCTCGAC	Gn1a frw	genotyping/editing check
OsP_1579	TCGAGCTCTAGCACGTTGG	Gn1a rev	genotyping/editing check
Osp_1580	AGCTGTTTGTGCAGAGCAAGT	Gs3 frw	genotyping/editing check
OsP_1581	ATTAACCAATCAGTGCAGCTTCG	Gs3 rev	genotyping/editing check
OsP_1582	TGTTTGCATTTGTGCTAATAGTGG	Gw2 frw	genotyping/editing check
OsP_1583	TGCCAACTGCCAAGGTTACA	Gw2 rev	genotyping/editing check
OsP_1584	TGATCGAGACAAACGGCGAA	pRGEB32 frw Cas9 screen	Cas9 check Xie et al., (2015)
OsP_1585	ACCAGCACAGAATAGGCCAC	pRGEB32 rev Cas9 screen	Cas9 check Xie et al., (2015)
OsP 1883	CATGAACCGCAACAAGTGGG	Gn1a frw	RT PCR
OsP_1884	ACCTCGCAGAAACCCAAGAT	Gn1a rev	RT PCR
OsP_1885	CACAGGGACATCGACCAGAAG	Gw2 frw	RT PCR
OsP_1886	ACTTGGGTAGTACAGGAAGCAG	Gw2 rev	RT PCR
OsP_1887	TCGGAAGAACTCCTGATCCAT	Gs3 frw	RT PCR
OsP_1888	GCACTTGCTCTGCACAAACA	Gs3 rev	RT PCR
Atp_5706	GTGAAGCTCAATAGAGAGGACC	Cas9 frw	pCas9 check Miao et al., (2013)
Atp_5718	CTTGATAATCTTGAGGAGGTCGTGG	Cas9 rev	pCas9 check Miao et al., (2013)
RPAD_G2	TCAAAACGACTTCCTCTGTG	*PROG1* deletion in O. glaberrima (from Wu et al. 2018)	*Kabre* genotyping
RPAD_G3	GGACAAAAGCATCTCCTCAA	*PROG1* deletion in O. glaberrima (from Wu et al. 2018)	*Kabre* genotyping
RM197_F	GATCCGTTTTTGCTGTGCCC	Size polymorphism between *O. sativa* and *O. glaberrima* (from Chen et al. 2017)	*Kabre* genotyping
RM197_R	CCTCCTCTCCGCCGATCCTG	Size polymorphism between *O. sativa* and *O. glaberrima* (from Chen et al. 2017)	*Kabre* genotyping
